# The Association between Nutritional Status and Malaria in Children from a Rural Community in the Amazonian Region: A Longitudinal Study

**DOI:** 10.1371/journal.pntd.0003743

**Published:** 2015-04-30

**Authors:** Márcia Almeida Araújo Alexandre, Silvana Gomes Benzecry, Andre Machado Siqueira, Sheila Vitor-Silva, Gisely Cardoso Melo, Wuelton Marcelo Monteiro, Heitor Pons Leite, Marcus Vinícius Guimarães Lacerda, Maria das Graças Costa Alecrim

**Affiliations:** 1 Gerência de Malaria, Fundação de Medicina Tropical Doutor Heitor Vieira Dourado, Manaus, Amazonas, Brazil; 2 Programa de Pós-graduação em Medicina Tropical, Universidade do Estado do Amazonas, Manaus, Amazonas, Brazil; 3 Department of Pediatrics, Discipline of Nutrition and Metabolism, Federal University of São Paulo, São Paulo, Brazil; 4 Laboratório de Pesquisa Clínica em Doenças Febris Agudas, Instituto Nacional de Infectologia Evandro Chagas, Fundação Oswaldo Cruz, Rio de Janeiro, Brazil; 5 Centro de Pesquisas Leônidas e Maria Deane, Fundação Oswaldo Cruz, Manaus, Amazonas, Brazil; Federal University of São Paulo, BRAZIL

## Abstract

**Background:**

The relationship between malaria and undernutrition is controversial and complex. Synergistic associations between malnutrition and malaria morbidity and mortality have been suggested, as well as undernutrition being protective against infection, while other studies found no association. We sought to evaluate the relationship between the number of malaria episodes and nutritional statuses in a cohort of children below 15 years of age living in a rural community in the Brazilian Amazon.

**Methodology/Principal Findings:**

Following a baseline survey of clinical, malaria and nutritional assessment including anthropometry measurements and hemoglobin concentration, 202 children ranging from 1 month to 14 years of age were followed for one year through passive case detection for malaria episodes. After follow-up, all children were assessed again in order to detect changes in nutritional indicators associated with malaria infection. We also examined the risk of presenting malaria episodes during follow-up according to presence of stunting at baseline. Children who suffered malaria episodes during follow-up presented worse anthropometric parameters values during this period. The main change was a reduction of the linear growth velocity, associated with both the number of episodes and how close the last or only malaria episode and the second anthropometric assessment were. Changes were also observed for indices associated with chronic changes, such as weight-for-age and BMI-for-age, which conversely, were more frequently observed in children with the last or only episode occurring between 6 and 12 months preceding the second nutritional assessment survey. Children with inadequate height-for-age at baseline (Z-score < -2) presented lower risk of suffering malaria episodes during follow-up as assessed by both the log-rank test (p =0.057) and the multivariable Cox-proportional hazards regression (Hazard Ratio = 0.31, 95%CI [0.10; 0.99] p=0.049).

**Conclusions:**

Malaria was associated with impaired nutritional status amongst children in an endemic area of the Western Brazilian Amazon where *P*. *vivax* predominates. Our data all supports that the association presents differential effects for each age group, suggesting distinct pathophysiology pathways. We were also able to demonstrate that undernourishment at baseline was protective to malaria during follow-up. These findings support an intriguing interaction between these conditions in the rural Amazon and the need for a more integrative approach by health systems in endemic areas.

## Author Summary

Malaria is one of the most serious public health problems in the world, with 3.3 billion people at risk of contracting the disease and almost one million deaths annually, primarily in children younger than five years of age. Undernutrition is also a morbidity of importance to the public worldwide and primarily affects children in tropical regions. In the present study, we sought to analyze the relationship between malaria and undernutrition in children living in a rural Amazonian community where malaria is endemic. Children from 1 month to 14 years of age were followed-up for 12 months through passive case detection (i.e., the presence of malarial parasites in peripheral blood in case of fever). Anthropometric and hemoglobin measurements and active malaria case detection tests (to detect asymptomatic parasitaemia) were conducted at the beginning and end of the follow-up period of 12 months. Children who had at least one episode of malaria during this period presented lower mean anthropometric index scores compared to children who did not have malaria. We concluded that malaria had a negative impact on the nutritional status of children living in an endemic area at the Western Brazilian Amazon, where *Plasmodium vivax* predominates.

## Introduction

Malaria is one of the most serious public health problems in the world, with 3.3 billion people at risk of contracting the disease and almost one million deaths annually, primarily in children under five years of age [1]. Although Latin American countries have experienced important reductions in malaria incidence, 490,545 cases were still reported in 2011, of which 363,948 (76.7%) were caused by *Plasmodium vivax*. Of these, 263,767 cases were reported in Brazil, mainly in the Amazon region, also with a vast majority of *P*. *vivax* (87.8%) [1, 2].

It has long been acknowledged that populations residing in malaria-endemic areas generally live under socio-economic conditions leading to poor nutritional status. The groups at highest risk for the adverse effects of malaria, children and pregnant women, are also those most affected by poor nutrition. Although it has been suspected that nutrition may influence susceptibility to the disease or alter its course, there have been comparatively few efforts to comprehensively examine such interactions [3, 4].

It has been gradually recognized that any infection is associated with a risk of worsening the nutritional status [5]. Whereas some studies have suggested that undernutrition is protective for presenting symptomatic malaria [4, 6], other studies have shown that undernutrition or the worsening of nutritional status may result in clinical complications and severe malaria by modifying the immune response [7–9]. On the other hand, some studies [10–12], have not been able to show a correlation between infection by *Plasmodium* and undernutrition [13, 14], with some studies even describing an antagonistic association between both entities [15].

The majority of studies addressing the relationship between malaria and nutritional status come from Africa, where *P*. *falciparum* is the main species causing malaria. In Latin America, there is scant information regarding the association between infectious diseases and nutrition. A survey performed in Central American countries showed that diarrhea and respiratory diseases worsened the nutritional status of children [5]. This nutritional impairment is most likely the result of multiple infections, poor socioeconomic status and inadequate diet, which in combination result in growth limitation. Previously conducted cross-sectional and case-control studies from Brazil have demonstrated an association between malaria and undernutrition in adults [16, 17]. In Colombia, a positive association was found between malaria and undernutrition prevalence in children [18, 19]. These studies however, do not allow for temporal analysis and a more conclusive causal association or the evaluation of dynamic parameters including growth velocity. In the Peruvian Amazon, children with vivax malaria disease were found to have a delay in linear growth and weight [20]. A recent study has found intriguing results of nutritional status and cognitive function after deworming in a malaria-endemic area in Cote d’Ivoire [21], with still unclear conclusion on the joint effect of helminths and malaria on nutritional status [22].

In the present study, we sought to analyze the occurrence of malaria episodes and undernutrition in a cohort of children living in a rural Amazonian community where malaria (caused by both *P*. *vivax* and *P*. *falciparum*) is endemic. The aim was to evaluate the effect of malaria episodes on the anthropometric nutritional status indicators change and to explore the influence of baseline height-for-age z-scores (HAZ), which more accurately reflects chronic undernutrition with less interference from recent episodes of malaria or other acute conditions) on the risk of subsequent malaria incidence.

## Methods

### Ethics statement

The study was approved by the Ethics Committee Board of the *Fundação de Medicina Tropical Dr*. *Heitor Vieira Dourado* (1899/2008 and 918/2010 approvals). Each participant and his/her parents or legal guardians signed written informed consent forms.

### Study area and population

The study was undertaken in two rural communities located in two recently colonized areas devoted to agriculture (Panelão and Castanho Sítio Communities), from May 2008 to May 2011. These settlements are located in the Municipality of Careiro, in the Amazonas State. The municipality has an area of 6,124,300 km^2^ and has 31,063 inhabitants. The climate is tropical and humid, with rainfalls ranging from 2,100 to 2,400 mm *per annum*. The municipality is connected to the capital of the state, Manaus, through a federal road (112 km of distance). Malaria is endemic in this area. The major economic activities are family farming, hunting and fishing. Drinking water comes from rainwater reservoirs or creeks. Garbage collection and sanitation are absent. Two health agents in each community are responsible for health care. The total population of both communities is 790 people, according to the census performed before the beginning of the study, including 300 children ranging from 1 month to 14 years of age.

**Fig 1:**
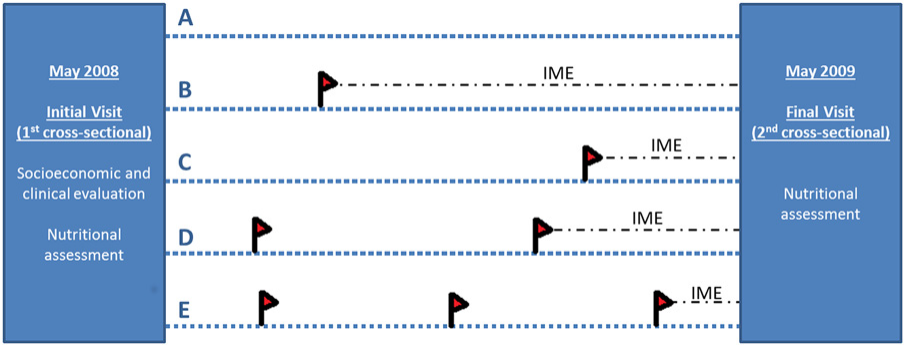
Study design illustrating five possible different patients. Initially a first visit was performed in which demographic and socioeconomic data was obtained and patients were submitted to a comprehensive clinical and nutritional assessment as well as microscopy examination of a blood sample (by thick smear?) for malaria infection detection. Patients were then followed for one year in which passive case detection for malaria episodes was performed in case of fever. At the end of the follow-up period patients were submitted to a final nutritional assessment. Illustrative scheme of possible patients (A-E) A Patient with no malaria episodes during follow-up (A); Patient with a single malaria episode occurring during the first semester of follow-up (B); Patient with a single malaria episode in the final stages of follow-up (C); Patient with two malaria episodes (D); Patient with three diagnosed malaria episodes (E). Red flag illustrating malaria episodes; IME—interval between the last malaria episode diagnosed during the follow-up period and the fnal visit assessment.

A cohort of 248 children ranging from 1 month to under 15 years of age was recruited for the present study, with 202 completing follow-up. As part of the baseline survey, a questionnaire was completed to collect socio-demographic information, including health history, maternal education, housing, and the number of assets (television sets, bicycles, fridge, motorcycle and cars). The housing characteristics were combined using principal component analysis (PCA) as previously described [23], in order to generate socioeconomic status indicators to classify individuals into one of three categories: the richest quintile, the three middle quintiles or the poorest quintile (Table 1). Children were recruited from May 2008 to May 2010. All children under 15 years of age were eligible and included if their guardians provided informed consent. Children lost to follow-up were excluded from the analysis. At the baseline survey, children were weighed and measured, and then subjected to thick blood smear, hemoglobin measurement and stool examination and were presumptively treated with mebendazole (100mg bid for 3 days) irrespectively of the stool results. Children were followed-up for 12 months through passive case detection of malaria based on microscopy in case of fever occurring at any time during the study. Anthropometric and hemoglobin measurements and thick blood smear (to diagnose asymptomatic parasitaemia) were repeated at the conclusion of the follow-up period, when no stool samples were repeated. Fig 1 illustrates the study design, showing 5 possible patients with different number/timing of malaria episodes.

**Table 1:** Baseline survey of 202 children who completed the follow-up in the Careiro municipality, Amazonas State.

Variable	n/ (%)
**Age (years)**	
Mean and standard deviation	7.8±3.4
< 5	39 (19.3)
5–10	108 (53.5)
>10–14	55 (27.2)
**Gender**	
M	109 (54)
F	93 (46)
**No. of family members**	
≤ 5	54 (26.7)
6–7	55 (27.2)
≥ 8	93 (46.1)
**Mother's education**	
Illiterate	19 (9.9)
Basic education	183 (91.1)
Higher education	0 (0)
**Socioeconomic status**	
Poorest quintile	81 (40.1)
Middle (2^nd^— 4^th^ quintiles)	90 (44.6)
**Previous malaria attacks**	
Yes	51 (25.2)
No	151 (74,8)
**Hemoglobin**	
Mean and standard deviation	11.6±1.6
< 11.9 g/dL	110 (54.5)
≥ 12 g/L	92 (45.5)
**Soil transmitted helminths**	
*Ascaris lumbricoides*	54 (26.7)
*Ancylostoma duodenale*	15 (7.4)
*Trichuris trichiura*	17 (8.4)
Protozoan parasites	
*Entamoeba histolytica/dispar*	15 (7.4)
*Giardia lamblia*	27(13.4)

To evaluate whether malaria affects nutritional status, the following outcome variables were used: growth velocity and the weight for height z-scores (WHZ), weight for age z-scores (WAZ), height for age z-scores (HAZ) and body mass index for age (BMI-Z) nutritional index scores, all of them considering the WHO standard cut-offs [24]. Malaria was ascertained by the presence of fever and positive parasitemia with additional reporting on the number of malaria episodes experienced by each individual and time between each of the episodes in relation to the first and second visits. Therefore we considered only disease caused by *Plasmodium* and did not systematically examined patients for detection of asymptomatic infection.

### Nutritional status assessment

Nutritional assessment through anthropometric measurements was performed during the cross-sectional evaluations, at the beginning and at the end of the follow-up. Anthropometric measurements were performed with minimal clothing and no shoes, according to guidelines by the Brazilian Ministry of Health [25]. Weight and height were obtained by internationally recommended methods [26]. Weight was measured using a digital weight balance for children above 2 years of age and a mechanical paediatric balance for younger children, both in the grams scale, while height was assessed by a single observer with the use of a portable anthropometer stadiometer with lateral scale in centimeters. All instruments were adjusted and verified after each measurement and fully calibrated every three months. The measurement techniques were harmonized according to the mentioned procedures [25–27], with all team members following the same standardized protocol.

Body mass index (BMI) was calculated using the program ANTHRO and ANTHRO PLUS [24]. BMI for age Z-scores below -2 were defined as undernutrition. Growth velocity was measured in cm/year, defined as the difference between the final and initial height in the period of 12 months. The classification of each individual as having adequate or inadequate growth was made according to the WHO standards for each age [28]

### Laboratory procedures

#### Malaria diagnosis

The thick blood smear (TBS) was prepared as recommended by the Walker technique [29] and evaluated by an experienced microscopist, who confirmed the species and determined the peripheral parasitemia, quantifying the asexual forms per 100 leukocytes counted in high-magnification fields using an estimate of 5,000 leukocytes/mm^3^ for each child. A sub-sample of blood collected in filter papers was also tested by PCR to allow quality control of the TBS for species-specific diagnosis (in total 138 samples were submitted to molecular testing, with a 97.8% concordance rate). A malaria case was considered when the child presented with fever and a positive TBS.

#### Hemoglobin concentration

Hemoglobin concentration was measured in venous blood using a portable photometer (HemoCue, Anglholm, Sweden) at the beginning and at the end of the follow-up.

#### Stool examination

Stool samples were taken only at baseline to examine the association between helminth infection and baseline anthropometric indicators. The stool samples were stored in flasks containing 10% formalin as preservative. Flasks were labeled with the patient’s name and date of collection and were kept at room temperature until the end of the month, when all the stool samples were examined. Spontaneous sedimentation [30] and centrifugal-flotation in zinc sulphate solution [31] methods were applied before the samples were analyzed by direct observation with a microscope for the detection of helminths and protozoans parasites using the direct observation, Kato-Katz, Faust and Hoffman methods. All tests were performed and evaluated by a single experienced examiner. Due to logistic impairments, stool samples were not taken at the end of follow-up.

### Statistical analysis

Children were categorized into three age groups for analyses, according to WHO guidelines for use of indicators WAZ, HAZ, WHZ and BMIZ [32]: ages below 5-years-of-age, between 5 and 10, and between 10 and 14 (for which WAZ and WHZ were not applied). In order to evaluate the impact of malaria on changes on the nutritional status, two main approaches were used. First, we evaluated the nutritional status at end of follow-up according to occurrence of any malaria episode, followed by a further categorization of malaria regarding number of episodes during the study period. In order to investigate the influence of timing of malaria episode on the impact over the anthropometric changes, we categorized the interval between the last or only malaria episode and the second measurement (IME in Fig 1). Chi-squared test comparing the proportion of children with adequate and inadequate status was performed for each anthropometric variable according to the different malaria exposure variables (presenting malaria, number of malaria episodes and timing of last malaria episode). Univariable and multivariable logistic regression were performed for each age group; the latter after adjustment with the a priori defined variables age, gender, maternal education, and socioeconomic status. This association was tested against the occurrence or not of malaria episodes during the study period, number of malaria episodes and malaria categorization of time from last or only malaria episode, providing odds ratios (OR) and Wald test p-values, with children with no malaria serving as reference in all analyses. Additionally, the interaction between number of malaria episodes and time from last or only episodes was tested for each variable of interest by evaluating the addition of a linear interaction term to the multivariable logistic regression models for each outcome of interest. A second analysis was performed considering the change on the Z-score of malaria indicators from the first to the second assessments according to malaria status using univariable and multivariable linear regression following the same approach as previously described. Statistical significance was considered if p<0.05

The association between stunting (height for age below -2 Z-scores) at baseline and the risk of development of malaria was assessed considering time from enrollment to the first or only malaria episode, using survival analysis techniques with inspection of the Kaplan-Meier survival curve, with performance of log-rank test and computation of adjusted Cox proportional hazard ratios [33]. This was the only index for which this association was analyzed as it is an indicator of chronic malnutrition and also for it being less likely to suffer influence of recent acute conditions (i.e. malaria and other acute febrile illnesses), which could interfere and confound the analysis. All analyses were performed in Stata v.13.1 (Statacorp, USA).

## Results

### Overall description and baseline assessment

**Fig 2:**
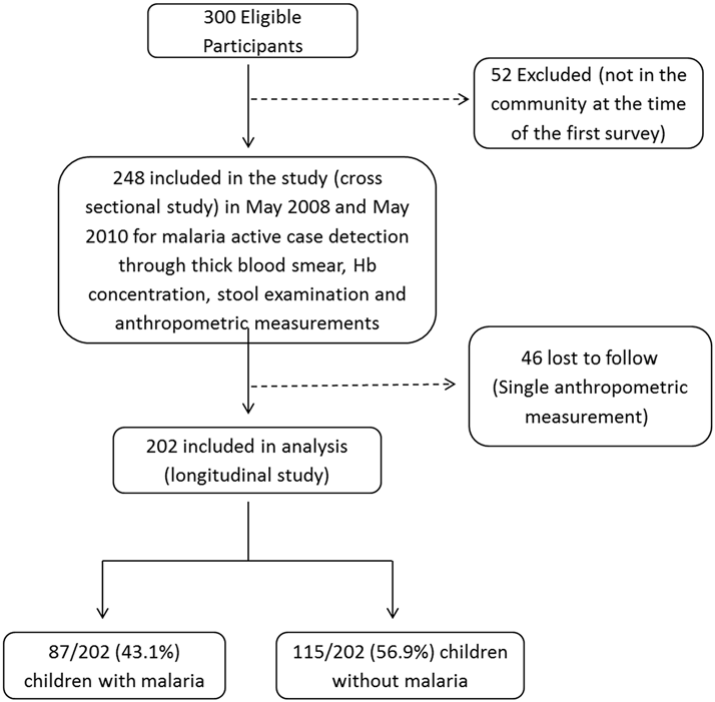
Study algorithm describing the details of eligible, enrolled and analysed children.

During the 12-month follow-up period, 248 of the 300 children eligible to participate in the cohort study were enrolled; 46 children were lost to follow up and the remaining 202 children were successfully followed (Fig 2). As shown in Table 1, most children included were between 5 and 10 years of age (53.5%), and a minority had experienced previous malaria episodes (25.2%). Hemoglobin below 11.9g/dL was observed in 110 children (54.5%) and the most common helminth parasite infecting children was *Ascaris lumbricoides* (26.7%). During the follow-up period, 87 children (43.1%) presented with at least one episode of malaria, and a total of 164 malaria episodes were observed. The remaining 115 children (56.9%) did not develop malaria during the study period. Among the infected subjects, 46 children (52.9%) had one malaria episode, 21 children (24.1%) had two malaria episodes, and 20 children (23%) had three or more malaria episodes. Regarding the *Plasmodium* species involved, 119 episodes (72.6%) consisted of mono infections with *P*. *vivax*, 37 episodes (22.5%) involved *P*. *falciparum* alone, and 8 episodes (4.9%) were mixed *P*. *vivax* and *P*. *falciparum* infections. No episodes of severe malaria occurred amongst the cohort participants.

Table 2 shows the nutritional status of the children at the baseline survey. In general, nutritional profile was similar amongst the three age groups. Children under 5 years of age showed a better nutritional status, considering WAZ and HAZ means scores. Frequencies of HAZ < -2 ranged from 7.7% in children under 5 to 10.9% in children between 10–14 years. Body mass index-z < -2 ranged from 1.9% in children 5–10 years of age to 7.7% in children under 5. The multivariable analyses considering age, gender and helminth infection could not identify any factor associated with inappropriate scores at baseline (S1 Table).

**Table 2:** Mean values and nutritional status classification of 202 participating children at the baseline survey.

Nutritional indicator		Age bands (years)	
	≤ 5 (n = 39)	5–10 (n = 108)	10–14 (n = 55)
	Mean (95%CI)	Mean (95%CI)	Mean (95%CI)
Weight-for-height Z-score (WHZ)	-0.26 (-0.61; 0.09)	-	-
Weight-for-age Z-score (WAZ)	-0.31 (-0.69; 0.11)	-0.50 (-0.69; -0.31)	-
Height-for-age Z-score (HAZ)	-0.10 (-0.80; 0.61)	-0.72 (-0.92; -0.53)	-0.94 (-1.29; -0.59)
BMI-for-age Z-score (BMI)	-0.28 (-0.63; 0.08)	-0.09 (-0.25; 0.08)	-0.35 (-0.62; -0.08)
	n (%)	n (%)	n (%)
WHZ < -2	0	- *	- *
WAZ < -2	0	0	- *
HAZ < -2	3 (7.7)	9 (8.3)	6 (10.9)
BMI-Z < -2	3 (7.7)	2 (1.9)	2 (3.6)

95%CI confidence interval for mean WHZ = Weight-for-height Z-score; WAZ = Weight-for-age Z-score; HAZ = Height-for-age Z-score; BMI-for-age Z = Body Mass Index-for-age Z-score.

* These scores are not used in these age groups.

### Influence of malaria episodes on anthropometric indicators

There was an association between final assessment being underweight, low BMI and inadequate growth velocity in children 5–10 years of age who developed malaria during follow-up (Table 3). Table 3 summarizes the results of the univariable and multivariable analysis evaluating nutritional profile according to malaria status. For 5–10 years-old children who had malaria [aOR 4.0 (95% CI 1.4; 11.4); p = 0.008] and a time of 6–12 months from the last malaria episode to the second nutritional assessment [aOR 4.4 (95% CI 1.3; 15.3); p = 0.020] there was a significant association with increased odds of inadequate growth velocity. In this age group, although with imprecise aORs, there is a trend of association between presenting one malaria episode [aOR 9.1 (95% CI 0.9; 85.8); p = 0.053] and a time of 6–12 months from the last malaria episode to the second nutritional assessment [aOR 8.4 (95% CI 0.9; 79.2); p = 0.062] and WAZ<-2, as there was for BMI-Z<-2 for having one malaria episode [aOR 7.5 (95% CI 0.8;73.0); p = 0.081] and a time of 6–12 months from the last malaria episode to the second nutritional assessment [aOR 6.9 (95% CI 0.7;65.9); p = 0.093].

**Table 3:** Univariable and multivariable analyses of the nutritional status on the second survey according to malaria status.

	WAZ†				HAZ				BMI-Z				Growth velocity			
	<-2 n (%)	p*	aOR (95%CI)	p¶	<-2n (%)	p*	aOR (95%CI)	p¶	<-2 n (%)	p*	aOR (95%CI)	p¶	<-2 n (%)	p*	aOR (95%CI)	p¶
**≤ 5 years**																
**Presented malaria (n)**																
**No (30)**	1 (3.3)	-	-	-	2 (6.7)	0.177	1		0	-	-		15 (50.0)		1	
**Yes (9)**	0	-	-	-	2 (22.2)		6.9 (0.3; 161.6)	0.229	0	-	-		4 (44.4)	0.770	1.1 (0.2; 6.4)	0.914
**5–10 years**																
**Presented malaria (n)**																
**No (58)**	1 (1.7)		1		8 (13.8)		1		1 (1.7)		1		34 (58.6)		1	
**Yes (50)**	4 (8.0)	0.122	5.0 (0.5; 45.9)	0.159	7 (14.0)	0.975	0.9 (0.3; 3.1)	0.923	4 (8.0)	0.122	4.2 (0.4; 39.1)	0.212	42 (84.0)	0.004	4.0 (1.4; 11.4)	0.008
**Number of malaria episodes (n)**																
**0 (58)**	1 (1.7)		1		8 (13.8)		1		1 (1.7)		1		34 (58.6)		1	
**1 (29)**	4 (13.8)	0.054	7.8 (0.8; 80.4)	0.079	4 (13.8)	0.442	0.9 (0.2; 3.7)	0.920	4 (13.8)	0.054	7.5 (0.8; 73.0)	0.081	21 (72.4)	0.005	2.1 (0.7; 6.2)	0.184
**2 (12)**	0		-		3 (25.0)		3.2 (0.5; 19.9)	0.222					12 (100)	-	-	
**≥ 3 (10)**	0		-		0		-						9 (100)	-	-	
**Time from last malaria to second assessment (n)**																
**No malaria (58)**	1 (1.7)		1		8 (13.8)		1		1 (1.7)		1		34 (58.6)		1	
**6–12 months (31)**	4 (12.9)	0.145	6.7 (0.7; 64.4)	0.101	5 (16.1)	0.941	1.2 (0.3; 4.2)	0.829	4 (12.9)	0.145	6.9 (0.7; 65.9)	0.093	26 (83.9)	0.064	4.4 (1.3; 15.3)	0.020
**3–6 months (15)**	0		-		2 (13.3)		0.8 (0.1; 4.5)	0.787	0		-		12 (80.0)		2.9 (0.7; 12.3)	0.151
**45–90 days (3)**	0		-		0		-		0		-		3 (100)		-	
**≤ 45 days (1)**	0		-		0		-		0		-		1 (100)		-	
**10–14 years**																
**Presented malaria (n)**																
**No (27)**	-		-		6 (22.2)		1		3 (11.1)		1		21 (77.8)		1	
**Yes (28)**	-		-		3 (10.7)	0.249	0.4 (0.1; 2.2)	0.290	2 (7.1)	0.609	1.0 (0.1; 17.0)	0.981	22 (78.6)	0.943	1.1 (0.2; 4.8)	0.930
**Number of malaria episodes (n)**																
**0 (27)**					6 (22.2)		1		3 (11.1)		1		21 (77.8)		1	
**1 (18)**					1 (5.6)	0.156	0.2 (0.01; 2.1)	0.159	2 (11.1)	0.748	2.0 (0.1; 36.7)	0.640	13 (72.2)	0.619	0.5 (0.1; 2.6)	0.389
**2 (5)**					0 (0.0)		-		0 (0.0)		-		4 (80.0)		1.3 (0.1; 19.8)	0.869
**≥ 3 (5)**					2 (40.0)		1.9 (0.2; 16.2)	0.562	0 (0.0)		-		5 (100.0)		-	
**Time from last malaria to second assessment (n)**																
**No malaria (27)**					6 (22.2)		1		3 (11.1)		1		21 (77.8)		1	
**6–12 months (20)**					1 (5.0)	0.392	0.2 (0.01; 1.7)	0.121	2 (10.0)	0.813	1.6 (0.1; 30.4)	0.735	15 (75.0)	0.738	0.8 (0.2; 3.8)	0.741
**3–6 months (4)**					1 (25.0)		1.9 (0.1; 28.3)	0.658	0 (0.0)		-		4 (100.0)		-	
**≤ 90 days (4)**					1 (25.0)		1.1 (0.1; 14.9)	0.956	0 (0.0)		-		3 (75.0)		1.1 (0.1; 14.6)	0.958

^†^WAZ was not measured for the 10–14 years-old group;

* P- value derived from chi-square test;

^¶^P-value derived from Wald test;

aOR—adjusted odds ratio from the multivariable logistic regression (adjusted for age, maternal education, socioeconomic status and gender); WHZ = Weight-for-height Z-score; WAZ = Weight-for-age Z-score; HAZ = Height-for-age Z-score; BMI-for-age Z = Body Mass Index-for-age Z-score.

No significant interaction was found between nutritional indicators at baseline and second measurements and malaria for children <5 year of age (Table 4). For the 5–10 years group, malaria was significantly associated with WAZ [adjusted β = -0.3 (-0.5;-0.1); p = 0.025)] and HAZ [adjusted β = -0.1 (-0.3;0.0); p = 0.035)]; one malaria episode was significantly associated with WAZ [adjusted β = -0.3 (-0.6;0.0); p = 0.024)] and BMI-Z [adjusted β = -0.6 (-1.1;-0.2); p = 0.005)]; two [adjusted β = -0.3 (-0.5;-0.1); p = 0.012)] or ≥3 [adjusted β = -0.3 (-0.5;0.0); p = 0.023)] malaria episodes were significantly associated with HAZ. A time of 6–12 months from the last malaria episode to the second nutritional assessment was significantly associated with WAZ [adjusted β = -0.4 (-0.7;-0.1); p = 0.006)] and BMI-Z [adjusted β = -0.6 (-1.0;-0.2); p = 0.006)]. For 10–14 years-old children, ≥3 malaria episodes was significantly associated with HAZ [adjusted β = -0.3 (-0.6;-0.1); p = 0.014)].

**Table 4:** Univariable and multivariable analyses of the between surveys difference in the anthropometric measurements according to malaria status.

	WAZ†				HAZ				BMI-Z			
	Unadjusted		Adjusted		Unadjusted		Adjusted		Unadjusted		Adjusted	
	β (95% CI)	p	β (95% CI)	p	β (95% CI)	p	β (95% CI)	p	β (95% CI)	p	β (95% CI)	p
**≤ 5 years**												
**Presented malaria (n)**												
No (30)	Ref		Ref		Ref		Ref		Ref		Ref	
Yes (9)	0.1 (-0.5; 0.6)	0.722	0.2 (-0.4; 0.8)	0.525	-0.2 (-1.3; 0.9)	0.765	-0.2 (-1.1; 1.2)	0.795	0.2 (-0.6; 1.0)	0.606	0.4 (-0.5; 1.2)	0.365
** 5–10 years**												
**Presented malaria (n)**												
** No (58)**												
** Yes (50)**	-0.3 (-0.5; -0.1)	0.018	-0.3 (-0.5; -0.1)	0.025	-0.2 (-0.3; -0.1)	0.011	-0.1 (-0.3; 0.0)	0.035	-0.3 (-0.7; 0.1)	0.126	-0.4 (-0.7; 0.0)	0.063
**Number of malaria episodes**												
** 0 (58)**	Ref		Ref		Ref		Ref		Ref		Ref	
** 1 (29)**	-0.4 (-0.6;-0.1)	0.012	-0.3 (-0.6; 0.0)	0.024	-0.1 (-0.3; 0.1)	0.012	0.0 (-0.2; 0.1)	0.517	-0.5 (-0.9; -0.1)	0.011	-0.6 (-1.1; -0.2)	0.005
** 2 (12)**	-0.1 (-0.5; 0.3)	0.701	0.0 (-05; 0.4)	0.952	-0.4 (-0.6;-0.1)	0.002	-0.3 (-0.5; -0.1)	0.012	0.2 (-0.3; 0.8)	0.426	0.2 (-0.4; 0.8)	0.531
** ≥ 3 (10)**	-0.3 (-0.7; 0.1)	0.155	-0.4 (-0.9; 0.1)	0.082	-0.3 (-0.5; -0.1)	0.046	-0.3 (-0.5; 0.0)	0.023	-0.1 (-0.8; 0.5)	0.711	-0.2 (-0.8; 0.4)	0.549
**Time from last malaria to second assessment (n)**												
No malaria (58)	Ref		Ref		Ref		Ref		Ref		Ref	
6–12 months (31)	-0.4 (-0.7; -0.2)	0.002	-0.4 (-0.7; -0.1)	0.006	-0.2 (-0.3; -0.1)	0.047	-0.1 (-0.3; 0.0)	0.102	-0.5 (-0.9; -0.1)	0.016	-0.6 (-1.0; -0.2)	0.006
3–6 months (15)	0.1 ((-0.3; 0.4)	0.608	0.0 (-0.4; 0.4)	0.961	-0.2 (-0.4; 0.04)	0.107	-0.1 (-0.3; 0.1)	0.145	0.2 (-0.4; 0.7)	0.536	0.1 (-0.4; 0.6)	0.717
45–90 days (3)	-0.5 (-1.2; 0.1)	0.081	-0.5 (-1.1; 0.2)	0.183	-0.4 (-0.9; 0.1)	0.080	-0.3 (-0.7; 0.1)	0.137	-0.4 (-1.4; 0.7)	0.512	-0.4 (-1.5; 0.7)	0.455
≤ 45 days (1)					-0.2 (-1.0; 0.5)	0.552	-0.1 (-0.8; 0.5)	0.669	0.3 (-1.5; 2.1)	0.758	0.1 (-1.7; 2.0)	0.881
**10–14 years**												
**Presented malaria (n)**												
Yes (27)					-0.1 (-0.3; 0.1)	0.102	-0.1 (-0.3; 0.1)	0.179	0.0 (-0.2; 0.2)	0.912	0.1 (-0.2; 0.3)	0.673
No (28)					Ref		Ref		Ref		Ref	
**Number of malaria episodes (n)**												
0 (27)												
1 (18)					-0.1 (-0.2; 0.1)	0.479	0.0 (-0.2; 0.2)	0.982	-0.1 (-0.3; 0.2)	0.685	0.0 (-0.3; 0.3)	0.894
2 (5)					-0.2 (-0.5; 0.1)	0.099	-0.3 (-0.5; 0.0)	0.058	0.2 (-0.2; 0.6)	0.369	0.4 (-0.1; 0.8)	0.124
≥ 3 (5)					-0.3 (-0.6; 0.1)	0.06	-0.3 (-0.6; -0.1)	0.014	-0.1 (-0.5; 0.4)	0.743	0.1 (-0.4; 0.5)	0.780
**Time from last malaria to second assessment (n)**												
No malaria (27)					Ref		Ref		Ref		Ref	
6–12 months (20)					-0.1 (-0.3; 0.1)	0.272	-0.1 (-0.2; 0.1)	0.492	-0.1 (-0.4; 0.2)	0.449	0.0 (-0.3; 0.3)	0.821
3–6 months (4)					-0.2 (-0.6; 0.1)	0.146	-0.2 (-0.5; 0.1)	0.176	0.2 (-0.3; 0.7)	0.403	0.1 (-0.3; 0.6)	0.519
≤ 90 days (4)					-0.2 (-0.5; 0.1)	0.207	-0.2 (-0.5; 0.1)	0.140	0.2 (-0.5; 0.7)	0.391	0.3 (-0.1; 0.8)	0.170

^†^WAZ was not measured for the 10–14 years-old group;

β—coefficient from the linear regression; 95% CI—95% confidence interval; ¶P-value derived from Wald test; WHZ = Weight-for-height Z-score; WAZ = Weight-for-age Z-score; HAZ = Height-for-age Z-score; BMI-for-age Z = Body Mass Index-for-age Z-score; multivariable analyses performed adjusting for age, maternal education, socioeconomic status and gender.

### Baseline nutritional status and risk of malaria episodes

**Fig 3:**
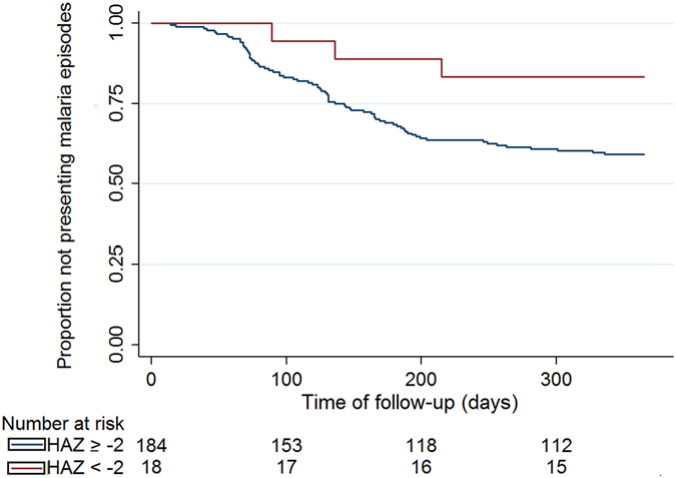
Kaplan-Meier survival analysis showing the time elapsed from the baseline to the first malarial infection (in days) according to baseline HAZ status.

The results of the analysis on the risk of malaria episodes related to the baseline nutritional status was assessed only for the or the HAZ indicator as this reflects chronic malnutrition more accurately and are shown on Fig 3. It was possible to observe a lower rate of malaria amongst children classified with low levels (Z-score < -2) on both the log-rank test (p = 0.057) and the multivariable Cox-proportional hazards regression (Hazard Ratio = 0.31, 95%CI [0.10; 0.99] p = 0.049). We have additionally examined the relationship between baseline anemia and risk of malaria episodes, with no evidence of an association (HR = 0.8; 95%CI = 0.5; 1.2; p = 0.239).

## Discussion

Malaria is one of the main public health problems in several developing countries affecting especially children, a particularly vulnerable population with the highest morbidity and mortality burdens associated with this disease [1, 34]. Malaria usually co-exists with other diseases and poor socioeconomic status, further impairing the development of the affected populations. Malnutrition is one of the most common and worrying conditions, impairing child development and the severity of other health conditions [35]. The concomitance of both conditions has been studied, but the mechanisms and clinical impacts of this association remain incompletely understood.

In this study, we show that malaria episodes interferes with the nutritional status of children, with notable reduced linear growth velocity as well as impairment in other indices associated with chronic malnutrition, even after adjustment for other factors. Suffering from one or multiple episodes of malaria had a significant negative effect on the linear growth velocity of children, especially amongst children between 5 and 10 years of age (aOR = 4.0; 95%CI: 1.4; 11.4, p = 0.008). This important finding suggests that even in areas where malaria transmission is low and *P*. *vivax* is the most prevalent species, the infection may influence the physical development of children. Lee et al. monitored a cohort of children in the Peruvian Amazon and observed that children with *P*. *vivax* malaria experienced delayed linear and weight growth [20]. Available reports indicating that malaria infection can trigger acute undernutrition [36, 37] and the effect of reducing malaria transmission on the improvement of undernutrition among children [38–40] are in agreement with our findings. We did not find an association for many of the investigated relationships, in particular for children below the age of 5. (a particularly vulnerable group for physical and developmental changes) due to limitations related to the low number of events during the study period. A trend of inadequate classification of WAZ and BMI-Z for children between 5 and 10 years of age presenting malaria was not observed, what could be related to specific developmental pathways being affected within this age range.

Although we found an association between undernourishment measured by HAZ and the risk of developing malaria, one needs to be careful at implying causality as other unmeasured confounders could be associated, including, for example, improved malaria preventive measures for ill children. We also did not observe a protective effect between baseline anemia and the risk of developing malaria as has been suggested elsewhere [41]. Due to the absence of severe episodes, we were not able to investigate the association of this condition with the anthropometric measurements.

Some studies investigating the pathophysiologic mechanisms suggest that in addition to the anorexia and vomiting caused by acute-phase malaria and the negative nitrogen balance during fever episodes [7], a lack of micronutrients, such as vitamin A and zinc, is a mechanism that can explain the effect of malaria on the nutritional status of infected children, particularly children younger than five years of age [42, 43]. After several malaria episodes, a delay in the physical development of these children may occur. Our findings provide further evidence from a specific endemic setting.

There were some important limitations in our study. Overall there was a low rate of malaria infection, especially amongst children in the lowest age group, which resulted in low power to examine the association of the infection with nutritional status and may, at least partially, explain the lack of effect observed amongst this particular group. Combined with the possibility of non-differential measurement error of the anthropometric indices, we assume the risk of bias towards a null effect of the association. No assessment of dietary patterns was performed, and although there is evidence of homogeneous habits within the study population, there may be important individual variations that would have a strong impact on the nutritional status of children. The lack of stool examination on the end of follow-up after the decision to treat all patients at inclusion has impaired the capacity to evaluate and control for the important influence that helminths have on the nutritional status of children [21, 22], especially in rural areas, what must undoubtedly be done in future studies examining this association. There would have been beneficial to the understanding of the causal mechanisms involved if we had systematically examined the children for asymptomatic infection, which could have had an important effect on our assessments, although this is assumed to be a rare finding amongst children of moderate transmission settings.

In summary, we found malaria to be associated with the impairment of nutritional status in children, with evidence pointing to specific effects for different age groups, as indicated by anthropometric indexes associated with chronic (BMI, HAZ) and acute (linear growth velocity) nutritional deficits. We were not able to establish a clear association between baseline anthropometric classification and the risk of developing malaria. Bigger cohorts may be necessary especially in areas of low malaria incidence, and future studies should focus on specific nutritional deficiencies that may influence *Plasmodium* infections. Undernutrition and malaria are important morbidities with relevance to public health; thus, coordinating the actions of malaria control programs and nutrition programs could substantially impact these morbidities among children, meriting further attention by researchers and policy makers.

## Supporting Information

S1 Checklist STROBE checklist. (DOCX)

S1 Dataset Dataset of the study used for the analyses. (RAR)

S1 Table Risk factors for inappropriate score (Z < -2) for HAZ and BAZ. (DOCX)
