# Supplementary material for: The Association between Nutritional Status and Malaria in Children from a Rural Community in the Amazonian Region: A Longitudinal Study
Source: PLoS Negl Trop Dis. 2015 Apr 30;9(4):e0003743. doi: 10.1371/journal.pntd.0003743 (PMC4415998; doi:10.1371/journal.pntd.0003743)
Supplement: S1 Checklist — (DOCX) [file pntd.0003743.s001.docx]

STROBE Statement—checklist of items that should be included in reports of observational studies

|  | Item No. | Recommendation | Page  No. | Relevant text from manuscript |
| --- | --- | --- | --- | --- |
| **Title and abstract** | 1 | (*a*) Indicate the study’s design with a commonly used term in the title or the abstract | 1-2 | The association between nutritional status and malaria in children from a rural community in the Amazonian region: a longitudinal study |
|  |  | (*b*) Provide in the abstract an informative and balanced summary of what was done and what was found | 2 |  |
| Introduction | | | |  |
| Background/rationale | 2 | Explain the scientific background and rationale for the investigation being reported | 5-6 |  |
| Objectives | 3 | State specific objectives, including any prespecified hypotheses | 6 | In the present study, we sought to analyze the frequency of malaria and undernutrition in a cohort of children living in a rural Amazonian community where malaria is endemic in order to investigate the effect of malaria on the anthropometric nutritional status indicators change and to explore the influence of baseline HAZ (which more accurately reflects chronic undernutrition with less interference from recent episodes of malaria or other acute conditions) on the risk of subsequent malaria incidence |
| Methods | | | |  |
| Study design | 4 | Present key elements of study design early in the paper | 7 | A cohort of 248 children ranging from 1 month to 14 years of age was recruited for the present study, with 202 completing follow-up |
| Setting | 5 | Describe the setting, locations, and relevant dates, including periods of recruitment, exposure, follow-up, and data collection | 7-11 |  |
| Participants | 6 | (*a*) *Cohort study*—Give the eligibility criteria, and the sources and methods of selection of participants. Describe methods of follow-up  *Case-control study*—Give the eligibility criteria, and the sources and methods of case ascertainment and control selection. Give the rationale for the choice of cases and controls  *Cross-sectional study*—Give the eligibility criteria, and the sources and methods of selection of participants | 7-11 |  |
|  |  | (*b*) *Cohort study*—For matched studies, give matching criteria and number of exposed and unexposed  *Case-control study*—For matched studies, give matching criteria and the number of controls per case | - |  |
| Variables | 7 | Clearly define all outcomes, exposures, predictors, potential confounders, and effect modifiers. Give diagnostic criteria, if applicable | 7-11 |  |
| Data sources/ measurement | 8* | For each variable of interest, give sources of data and details of methods of assessment (measurement). Describe comparability of assessment methods if there is more than one group | 9 | *Body mass index (BMI) was calculated using the program ANTHRO® and ANTHRO PLUS® [23]. BMI Z-scores < -2 were defined as malnutrition; Z-scores below -3 were defined as severe malnutrition; Z-scores between -1 and 2 were defined as normal weight; and Z-scores >2 were defined as obesity. Growth velocity was measured in cm/year. The growth velocity is defined as the difference between the final and initial height in the period of 12 months, and the adequacy to each individual was established according to the WHO standards [24]* |
| Bias | 9 | Describe any efforts to address potential sources of bias | - |  |
| Study size | 10 | Explain how the study size was arrived at | 7 | The total population of both communities is 790 people, according to the census performed before the beginning of the study, including 300 children ranging from 1 month to 14 years of age. |

Continued on next page

| Quantitative variables | 11 | Explain how quantitative variables were handled in the analyses. If applicable, describe which groupings were chosen and why |  |  |
| --- | --- | --- | --- | --- |
| Statistical methods | 12 | (*a*) Describe all statistical methods, including those used to control for confounding | 10 | Children were categorized into three age groups for analyses, according to WHO guidelines for use of indicators W/A, H/A, W/H and BMI [28]: ages below 5-years-of-age, between 5 and 10, and between 10 and 14 (for which W/A and W/H were not applied). In order to evaluate the impact of malaria on changes on the nutritional status, two main approaches were used. First, we evaluated the nutritional status according to occurrence of any malaria episode, followed by a further categorization of malaria regarding number of episodes during the study period. In addition, in order to investigate the duration and causality of malaria impact, we also evaluated the timing between the last or only malaria episode and the second measurement. For this purpose univariable and multivariable logistic regression were used for each age group, the latter after adjustment with the a priori defined variables age, gender, maternal education, presence of soil transmitted helminth and socioeconomic status. This association was tested against the occurrence or not of malaria episodes during the study period, number of malaria episodes and malaria categorization of time from last or only malaria episode. Additionally, the synergy between number of malaria episodes and time from last or only episodes was tested for each variable of interest by evaluating the addition of a linear interaction term to the multivariable logistic regression models for each outcome of interest. A second analysis was performed considering the change on the Z-escore of malaria indicators from the first to the second assessments according to malaria status using univariable and multivariable linear regression following the same approach as previously described.  The association between HAZ baseline classification (categorizing patients as stunting if the Z-score was below -2) and the risk of development of malaria episodes, survival analysis techniques were used with assessment through the K-M graph and multivariable Cox proportional hazards regression adjusted for the same variables previously described. This was the only index for which this association was analyzed as it is an indicator of chronic malnutrition and also for it being less likely to suffer influence of recent acute conditions (i.e. malaria and other acute febrile illnesses), which could interfere and confound the analysis. All analyses were performed in Stata® v.13.1 (Statacorp®, USA). |
|  |  | (*b*) Describe any methods used to examine subgroups and interactions |  | 10 |
|  |  | (*c*) Explain how missing data were addressed |  | - |
|  |  | (*d*) *Cohort study*—If applicable, explain how loss to follow-up was addressed  *Case-control study*—If applicable, explain how matching of cases and controls was addressed  *Cross-sectional study*—If applicable, describe analytical methods taking account of sampling strategy |  | 10 |
|  |  | (*e*) Describe any sensitivity analyses |  | 10 |
| Results | | | | |
| Participants | 13* | (a) Report numbers of individuals at each stage of study—eg numbers potentially eligible, examined for eligibility, confirmed eligible, included in the study, completing follow-up, and analysed | 12 | During the 12-month follow-up period, 248 of the 300 children eligible to participate in the cohort study were enrolled; 46 children were lost to follow up and the remaining 202 children were successfully followed (Figure 2). |
|  |  | (b) Give reasons for non-participation at each stage |  | 12 |
|  |  | (c) Consider use of a flow diagram |  | Yes – figura2 |
| Descriptive data | 14* | (a) Give characteristics of study participants (eg demographic, clinical, social) and information on exposures and potential confounders | 12 | As shown in table 1, most children included were between 5 and 10 years of age, and a minority had experienced previous malaria episodes. During the follow-up period, 87 children (43.1%) presented with at least one episode of malaria, and a total of 164 malaria episodes were observed. The remaining 115 children (56.9%) did not develop malaria during the study period. Among the infected subjects, 46 children (52.9%) had one malaria episode, 21 children (24.1%) had two malaria episodes, and 20 children (23%) had three or more malaria episodes. Regarding the Plasmodium species involved in these episodes, 119 episodes (72.6%) consisted of mono infections with P. vivax, 37 episodes (22.5%) involved P. falciparum alone, and 8 episodes (4.9%) were mixed P. vivax and P. falciparum infections |
|  |  | (b) Indicate number of participants with missing data for each variable of interest |  | - |
|  |  | (c) *Cohort study*—Summarise follow-up time (eg, average and total amount) |  | 12 |
| Outcome data | 15* | *Cohort study*—Report numbers of outcome events or summary measures over time |  | *-* |
|  |  | *Case-control study—*Report numbers in each exposure category, or summary measures of exposure |  |  |
|  |  | *Cross-sectional study—*Report numbers of outcome events or summary measures |  |  |
| Main results | 16 | (*a*) Give unadjusted estimates and, if applicable, confounder-adjusted estimates and their precision (eg, 95% confidence interval). Make clear which confounders were adjusted for and why they were included |  | - |
|  |  | (*b*) Report category boundaries when continuous variables were categorized |  | - |
|  |  | (*c*) If relevant, consider translating estimates of relative risk into absolute risk for a meaningful time period |  | - |

Continued on next page

| Other analyses | 17 | Report other analyses done—eg analyses of subgroups and interactions, and sensitivity analyses | 12-13 |  |
| --- | --- | --- | --- | --- |
| Discussion | | | | |
| Key results | 18 | Summarise key results with reference to study objectives | 13 |  |
| Limitations | 19 | Discuss limitations of the study, taking into account sources of potential bias or imprecision. Discuss both direction and magnitude of any potential bias | 15 | There were some important limitations in our study |
| Interpretation | 20 | Give a cautious overall interpretation of results considering objectives, limitations, multiplicity of analyses, results from similar studies, and other relevant evidence | 15-16 |  |
| Generalisability | 21 | Discuss the generalisability (external validity) of the study results | - |  |
| Other information | |  | | |
| Funding | 22 | Give the source of funding and the role of the funders for the present study and, if applicable, for the original study on which the present article is based | Yes (no system) |  |

*Give information separately for cases and controls in case-control studies and, if applicable, for exposed and unexposed groups in cohort and cross-sectional studies.

**Note:** An Explanation and Elaboration article discusses each checklist item and gives methodological background and published examples of transparent reporting. The STROBE checklist is best used in conjunction with this article (freely available on the Web sites of PLoS Medicine at http://www.plosmedicine.org/, Annals of Internal Medicine at http://www.annals.org/, and Epidemiology at http://www.epidem.com/). Information on the STROBE Initiative is available at www.strobe-statement.org.
