# Supplementary material for: The Association between Nutritional Status and Malaria in Children from a Rural Community in the Amazonian Region: A Longitudinal Study
Source: PLoS Negl Trop Dis. 2015 Apr 30;9(4):e0003743. doi: 10.1371/journal.pntd.0003743 (PMC4415998; doi:10.1371/journal.pntd.0003743)
Supplement: S1 Table — (DOCX) [file pntd.0003743.s003.docx]

**S1 Table – Risk factors for inappropriate score (Z < -2) for HAZ and BAZ**

|  | HAZ | | BAZ | |
| --- | --- | --- | --- | --- |
|  | aOR (95%CI) | p | aOR | p |
|  |  |  |  |  |
| Helminth infection | 0.64 (0.2 – 2.1) | 0.458 | 0.38 (0.1-3.4) | 0.386 |
| Female gender | 1.35 (0.5-3.7) | 0.557 | 3.00 (0.5-16.4) | 0.206 |
| aOR – adjusted odds ratio from the multivariable logistic regression (adjusted for age, maternal education, socioeconomic status, gender and helminth infection); HAZ= Height-for-age Z-score; BMI-for-age Z =Body Mass Index-for-age Z-score. | | | | |
